# Perioperative management of spinal cord stimulators in patients undergoing non-SCS spine surgery: A retrospective cohort study

**DOI:** 10.1016/j.inpm.2026.100823

**Published:** 2026-09-14

**Authors:** Ahmad R. Saleh, Emily Standage, Choopong Luansritisakul, Eileen Jin, Matthew Robert Smith, David Hao

**Affiliations:** a Department of Emergency Medicine, NYP Weill Cornell Medical Center, New York, NY, USA; b Department of Internal Medicine, Mass General Brigham, Boston, MA, 02114, USA; c Harvard Medical School, USA; d Department of Anesthesiology, Faculty of Medicine Siriraj Hospital, Mahidol University, Bangkok, Thailand; e Department of Anesthesiology, Mass General Brigham, Boston, MA, 02114, USA

**Keywords:** Spinal cord stimulation, Neuromodulation, Perioperative care, Electrocoagulation, Patient safety, Retrospective studies

## Abstract

**Background:**

Spinal cord stimulation (SCS) use has expanded, so perioperative teams increasingly encounter these devices during unrelated spine surgery, where leads, anchors, and the generator may lie within or adjacent to the operative field. Real-world perioperative management of these devices remains uncharacterized.

**Objective:**

To characterize documented perioperative SCS management in adults undergoing non-SCS spine surgery and to benchmark prespecified management elements against current practical guidance.

**Methods:**

In this single-center retrospective pilot cohort study, we identified adults (≥18 years) with a previously implanted SCS who underwent non-SCS spine surgery between April 1, 2016, and March 22, 2026. Prespecified primary outcomes were alignment with (1) preoperative deactivation or surgery mode and (2) appropriate electrocautery management (bipolar only, or monopolar with the generator contralateral to the return pad). Secondary outcomes were device-related adverse events. Analyses were descriptive, using frequencies and Wilson 95% confidence intervals (CIs).

**Results:**

Fifty-eight surgical cases were included (mean age 59.2 [SD 13.8] years; 30 [51.7%] male; 42 [72.4%] instrumented fusion; all general anesthesia). Electrocautery was used in every case. Alignment with Element 1 was 13.8% (95% CI, 7.2 to 24.9) and to Element 2 was 43.1% (95% CI, 31.2 to 55.9); only 2 cases (3.4%; 95% CI, 1.0 to 11.7) met both. A perioperative plan was documented in 36.2% (95% CI, 25.1 to 49.1) and reactivation before discharge in 19.0% (95% CI, 10.9 to 30.9). Five cases (8.6%; 95% CI, 3.7 to 18.6) had an SCS-related adverse event, including one generator failure requiring replacement after monopolar cautery with an undocumented return-pad location.

**Conclusion:**

Documented perioperative SCS management during non-SCS spine surgery was infrequent and fell short of current guidance in this pilot study, coexisting with a small but non-trivial rate of device-related adverse events. These gaps warrant prospective evaluation of structured protocols and defined pain-service engagement.

## 1 Introduction

Spinal cord stimulation (SCS) use has expanded substantially over the past decade. In the fee-for-service Medicare population, SCS implants rose roughly 200% between 2009 and 2018 [1], and commercial claims data show SCS trialing increasing from 17.9 to 22.9 per 100,000 covered lives between 2010 and 2022 [2]. As the implanted population grows, perioperative teams are increasingly likely to encounter these devices during unrelated operations [[3], [4], [5]].

Non-SCS spine surgery is a particularly high-consequence setting: electrocautery near implanted hardware can damage or inactivate the system, the surgical field may overlie leads or anchors, and postoperative reactivation must be coordinated [3,5,6]. Recent guidance and manufacturer clinician manuals recommend early device identification, preincision deactivation or placement in surgery mode, preferential use of bipolar electrocautery, and return-pad positioning that keeps the anticipated current path away from the generator and leads when monopolar cautery is unavoidable [5,[7], [8], [9], [10]] Real-world alignment during non-SCS spine surgery has not been characterized. We therefore performed a retrospective cohort study of adults with previously implanted SCS undergoing non-SCS spine surgery, characterizing documented perioperative management and benchmarking prespecified elements against this guidance [5].

## 2 Methods

**Fig. 1:**
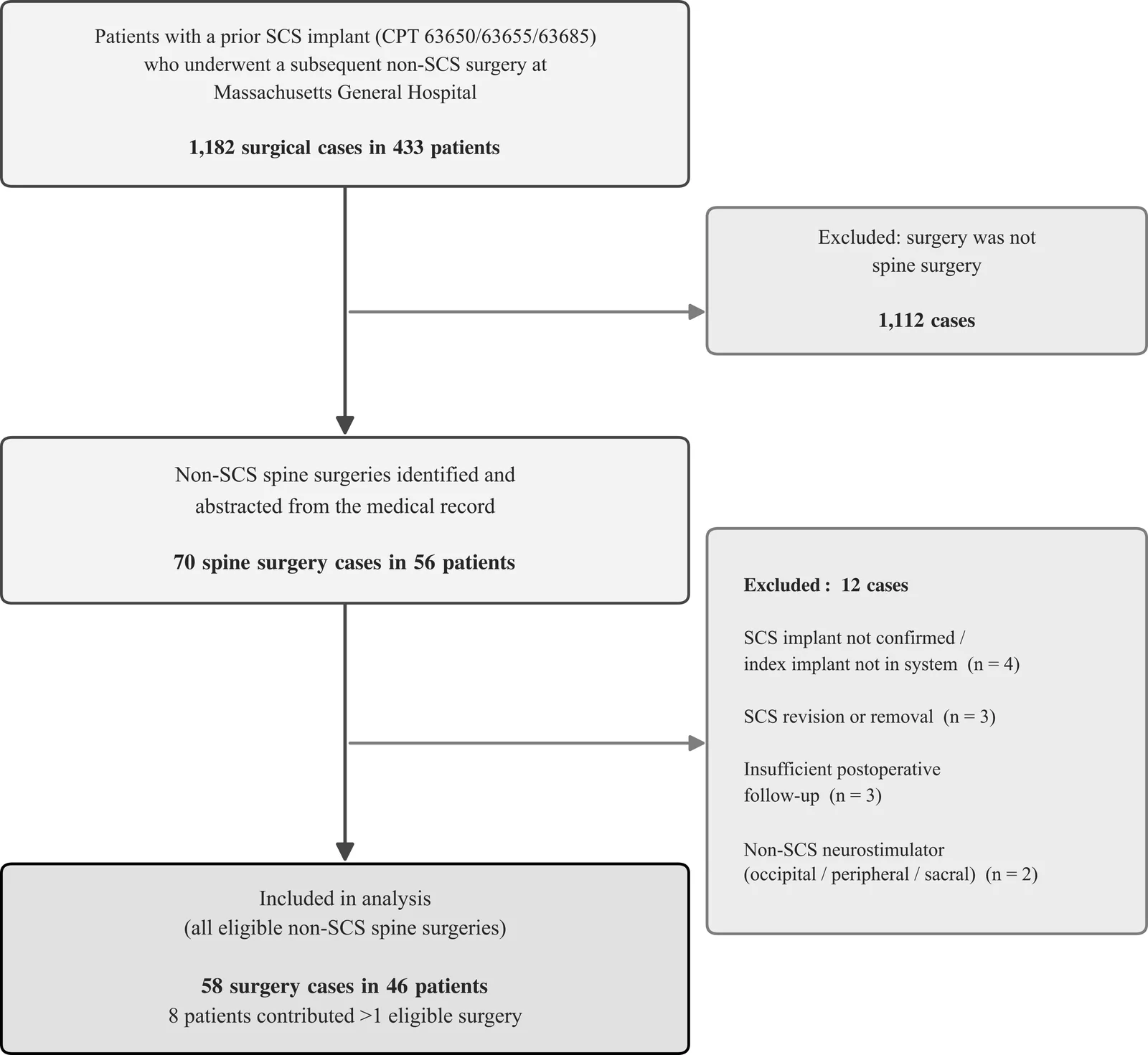
Cohort selection. Flow diagram of case selection for the study. Adults with a previously implanted spinal cord stimulator (SCS; CPT 63650/63655/63685) who underwent a subsequent non-SCS surgery were identified, then restricted to non-SCS spine surgery. Cases were excluded for SCS implant not confirmed or index implant not in the system (n = 4), SCS revision or removal (n = 3), insufficient postoperative follow-up (n = 3), and non-SCS neurostimulator, e.g. occipital, peripheral, or sacral (n = 2). The final cohort comprised 58 surgical cases in 46 patients, of whom 8 contributed more than 1 eligible surgery. SCS = spinal cord stimulation.

This retrospective cohort pilot study was approved by the Mass General Brigham Institutional Review Board (protocol 2025P003416) with a waiver of informed consent and follows the STROBE reporting guideline. We included adults (at least 18 years) with a previously implanted SCS who underwent non-SCS spine surgery at Massachusetts General Hospital between April 1, 2016 and March 22, 2026. We excluded patients whose device had been explanted before the surgery, procedures involving operation on an implanted pain device (including SCS or intrathecal pump), and records lacking an operative note (Fig. 1). Of the 46 included patients, 38 contributed a single surgery and 8 contributed more than one (range, 2–4); the surgical case was the unit of analysis. Patients who underwent multiple surgeries during the study period were treated as independent observations because each surgical procedure represented a distinct perioperative episode with its own perioperative management.

Trained reviewers abstracted records using a structured instrument and coding manual. Abstracted variables included patient and procedural characteristics and SCS device characteristics. For perioperative SCS management, reviewers abstracted whether a perioperative management plan was documented in the surgical preoperative note and, separately, in the anesthesia preoperative note; whether the device was documented as turned off preoperatively; and whether, for Abbott/St. Jude systems, it was documented as placed in surgery mode preoperatively. Reviewers also abstracted preoperative pain service or implanting physician consultation; anesthetic type and whether neuraxial anesthesia was performed; electrocautery use and type; IPG and return-pad location; documented postoperative device reactivation; and device-related events. Records were independently abstracted by three trained reviewers using a structured instrument and coding manual, and discrepancies were resolved by adjudication with the senior author.

The primary outcomes were alignment with two prespecified perioperative management elements.

Element 1 was preoperative device deactivation or surgery mode. A case was adherent if either the preoperative plan documented (in the surgical or anesthesia preoperative note) that the device would be turned off or, for Abbott/St. Jude systems, placed in surgery mode before incision; otherwise, it was nonadherent.

Element 2 was intraoperative electrocautery management. Cases in which no electrocautery was used were classified as not applicable and excluded. Bipolar-only electrocautery was considered adherent. Monopolar electrocautery, alone or combined with bipolar, was considered adherent only when the documented implantable pulse generator (IPG) location was contralateral to the documented return-pad location, such that the anticipated current path would not traverse the IPG; ipsilateral or same-side placement was nonadherent. Absence of a documented return-pad location in a monopolar case was classified as nonadherent in the primary analysis.

Secondary outcomes were device-related adverse events across four windows (intraoperative, before discharge, within 30 days, and 31 to 180 days after surgery), with abstraction of whether an intervention was required and whether the device was revised or explanted. Device-related events were additionally classified by plausible mechanism as either (1) potentially related to perioperative device management, including electrocautery exposure, failure to deactivate or enable surgery mode, or (2) attributable to the surgical procedure itself, in which hardware lying within or adjacent to the operative field was encountered, displaced, or exposed during dissection. This classification was assigned by consensus and is descriptive.

Analyses were descriptive. We report frequencies (percentages) with 95% confidence intervals (Wilson) for documentation and complication proportions, and mean (SD) for normally distributed continuous variables. No sample-size calculation was performed; all eligible surgeries during the study period were included. Analyses were performed in SPSS Statistics version 31.0.1.0 (IBM Corp., Armonk, NY, USA).

## 3 Results

Fifty-eight surgical cases (in 46 patients) met inclusion criteria (Table 1). Mean age was 59.2 (13.8) years, 30 (51.7%) were male, and most were ASA physical status III (39; 67.2%). Surgery was elective in 56 cases (96.6%) and was performed by neurosurgery in 32 (55.2%) and orthopedic surgery in 26 (44.8%). The lumbar spine was the most frequent operative region (29; 50.0%), and 42 procedures (72.4%) involved instrumented spinal fusion. All patients received general anesthesia. Patients in this cohort were cared for by a mixed group of general anesthesiologists; the specific subspecialty breakdown was not available, though few clinicians had chronic pain subspecialty training.

**Table 1:** Demographics and clinical characteristics of the study cohort.

Characteristics	N = 58
**Age, years**	59.2 (13.8)
**Sex (male)**	30 (51.7%)
**Body weight, kilograms**	88.4 (20.3)
**ASA physical status classification**	
II	19 (32.8%)
III	39 (67.2%)
**Urgency of surgery**	
Elective	56 (96.6%)
Urgent	1 (1.7%)
Unknown	1 (1.7%)
**Surgical specialty**	
Neurosurgery	32 (55.2%)
Orthopedics	26 (44.8%)
**Level of surgery**	
Cervical	11 (19.0%)
Cervicothoracic	4 (6.9%)
Thoracolumbar	3 (5.2%)
Lumbar	29 (50.0%)
Lumbosacral	10 (17.2%)
Thoracolumbosacral	1 (1.7%)
**Procedure type**	
Decompression alone	16 (27.6%)
Instrumented spinal fusion	42 (72.4%)
**Anesthetic technique**	
General anesthesia	58 (100%)

Continuous variables are presented as mean (standard deviation), and categorical variables are presented as frequency (percentage).

**Abbreviation:** ASA = American Society of Anesthesiologists.

Device characteristics are summarized in Table 2. The most common indication for SCS was persistent spinal pain syndrome type 2 (40; 69.0%). Most systems used percutaneous dorsal column leads (52; 89.7%) positioned at the thoracic levels (53; 91.4%); the predominant manufacturers were Medtronic (25; 43.1%) and Nevro (18; 31.0%).

**Table 2:** Neuromodulation device profile.

Device characteristics	N = 58
**Indication for SCS**	
PSPS type 1	12 (20.7%)
PSPS type 2	40 (69.0%)
CRPS	5 (8.6%)
Other^a^	1 (1.7%)
**Electrode type**	
Percutaneous dorsal column lead	52 (89.7%)
Percutaneous DRG lead	2 (3.4%)
Paddle lead	3 (5.2%)
Combined percutaneous dorsal column and paddle leads	1 (1.7%)
**Level of electrode**	
Cervical	2 (3.4%)
Thoracic	53 (91.4%)
Lumbar/Sacral	2 (3.4%)
Multiple	1 (1.7%)
**Device manufacturer**	
Abbott/St. Jude Medical	10 (17.2%)
Boston Scientific	2 (3.4%)
Medtronic	25 (43.1%)
Nevro	18 (31.0%)
Other^b^	3 (5.2%)

Data are presented as frequency (percentage).

**Abbreviations**: CRPS = complex regional pain syndrome, DRG = dorsal root ganglion, PSPS = persistent spinal pain syndrome, SCS = spinal cord stimulation.

a Other indications include spinal cord injury-related pain.

b Other manufacturers include Nuvectra and Saluda.

Documented alignment with the two prespecified elements was low (Table 3). Alignment with Element 1 (a documented preoperative plan for device deactivation or surgery mode) was observed in 8 cases (13.8%; 95% CI, 7.2 to 24.9%). A perioperative SCS management plan of any type was documented in the surgical or anesthesia preoperative note in 21 cases (36.2%; 95% CI, 25.1 to 49.1%), and preoperative pain service or implanting physician consultation in 5 (8.6%; 95% CI, 3.7 to 18.6%). Electrocautery was used in all cases (bipolar only, 8 (13.8%); monopolar only, 6 (10.3%); both bipolar and monopolar, 42 (72.4%); unclear, 2 (3.4%)). Among these, Element 2 (appropriate intraoperative electrocautery management) was met in 25 cases (43.1%; 95% CI, 31.2 to 55.9%). Postoperatively, device reactivation before discharge was documented in 11 cases (19.0%; 95% CI, 10.9 to 30.9%) and pain service consultation in 7 (12.1%; 95% CI, 6.0 to 22.9%). Only 2 cases (3.4%; 95% CI, 1.0 to 11.7%) were adherent to both elements.

**Table 3:** Alignment with perioperative recommendations for patients with spinal cord stimulators.

Recommendation	Alignment rate, n/N (%)	95% CI (%)
**1. Preoperative period**		
1.1 Preoperative pain service or implanting physician consultation	5/58 (8.6%)	3.7 to 18.6
1.2 Documentation of perioperative management plan of SCS in anesthesia or surgical preoperative note	21/58 (36.2%)	25.1 to 49.1
1.3 Documentation of device deactivation or activation of “Surgery Mode” (Abbott/St. Jude Medical devices only)	8/58 (13.8%)	7.2 to 24.9
**2. Intraoperative period**		
2.1 Appropriate intraoperative electrocautery management	25/58 (43.1%)	31.2 to 55.9
**3. Postoperative period**		
3.1 Documentation of device reactivation prior to hospital discharge	11/58 (19.0%)	10.9 to 30.9
3.2 Pain service consultation prior to discharge	7/58 (12.1%)	6.0 to 22.9
**Adherent to both recommendations 1.3 and 2.1**	2/58 (3.4%)	1.0 to 11.7

Five cases (8.6%; 95% CI, 3.7 to 18.6%) experienced an SCS-related adverse event (Table 4). Four were classified as attributable to the surgical procedure or of indeterminate relation to device management: intraoperative lead dislodgment during dissection, intraoperative hardware exposure, lead displacement identified after postoperative reactivation and managed by reprogramming, and delayed IPG charging difficulty in which no hardware fault was identified. Three of these occurred during revision or instrumented fusion procedures in which SCS hardware was in close proximity to the operative field, a recognized risk of spine surgery independent of perioperative device management. One case was classified as potentially related to perioperative device management: after monopolar electrocautery with an undocumented return-pad location, the IPG was damaged, could not be reactivated, and required surgical revision for generator replacement.

**Table 4:** Clinical details of patients experiencing perioperative SCS-related adverse events.

Case no.	Age, sex	ASA	Surgery	Anesthesia	Indication for SCS	Electrode type	Location of leads and IPG	Manufacturer	Perioperative management of SCS	Complication	Management and outcomes
**SCS-related adverse events during the intraoperative period**											
1	80, F	III	Posterior spinal fusion L1–pelvis with Smith–Petersen osteotomy at L3–4	G A	PSPS type 2	Percutaneous dorsal column	T7 (top); T7 (mid), IPG at right iliac fossa	Boston Scientific	- No preoperative pain service or implanting physician consultation - No documented perioperative SCS management plan - No documented device deactivation - Both monopolar and bipolar electrocautery were used - Grounding pad was placed on the left outer thigh	Intraoperative lead dislodgement during dissection	SCS device was removed during surgery. The patient underwent SCS reimplantation 9 months later.
2	53, M	III	Revision L2–pelvic posterior spinal fusion	GA	PSPS type 2	Percutaneous dorsal column	T7 (top), IPG at left iliac fossa	Nuvectra	- No preoperative pain service or implanting physician consultation - No documented perioperative SCS management plan - No documented device deactivation - Both monopolar and bipolar electrocautery were used - Grounding pads were placed on the bilateral posterior thighs	SCS hardware exposed intraoperatively; no documented intraoperative device damage or explantation.	No perioperative intervention required; however, the patient underwent staged SCS revision 10 months later.
**SCS-related adverse events prior to discharge**											
3	46, M	II	Revision T12-L3 fusion and L2-3 discectomy	GA	PSPS type 2	Percutaneous dorsal column	T8 (top); T9 (top), right iliac fossa	Nevro	- No preoperative pain service or implanting physician consultation - No documented perioperative SCS management plan - No documented device deactivation - Both monopolar and bipolar electrocautery were used - Grounding pads were placed on the bilateral outer thighs	SCS leads encountered intraoperatively; no documented intraoperative device damage or explantation. Following postoperative device reactivation, the patient reported a shock-like sensation in the right lower rib area; subsequent radiography demonstrated right lead displacement.	SCS device was reprogrammed to avoid right-sided stimulation.
**SCS-related adverse events within 30 days after surgery**											
4	70, M	III	Revision L3-4 laminectomy with right discectomy	GA	CRPS	Percutaneous DRG	L4 right, L5 right, S1 right, IPG at right iliac fossa	Abbott/St. Jude Medical	- No preoperative pain service or implanting physician consultation - SCS perioperative management plan documented - Device documented as turned off preoperatively, but not placed in Surgery Mode - Monopolar electrocautery was used - The location of grounding pad was not documented	SCS device could not be reactivated because the IPG was damaged by electrocautery during surgery.	SCS revision required for IPG replacement.
**SCS-related adverse events between 31-180 days after surgery**											
5	70, F	III	Right lateral T12-L1 and L1-L2 interbody fusion	GA	PSPS type 2	Percutaneous dorsal column	T8 (top); T8 (mid), IPG at right iliac fossa	Medtronic	- Pain physician consulted preoperatively - SCS perioperative management plan documented - No documented device deactivation - Both monopolar and bipolar electrocautery were used - Grounding pad was placed on the left outer thigh	IPG charging difficulty	SCS device was evaluated, and no hardware problems were identified. The patient was then reassured.

**Abbreviations:** CRPS = complex regional pain syndrome, DRG = dorsal root ganglion, F = female, GA = general anesthesia, IPG = implantable pulse generator, M = male, PSPS = persistent spinal pain syndrome, SCS = spinal cord stimulation.

## 4 Discussion

To our knowledge, this is the first study to characterize documented perioperative management of spinal cord stimulators specifically in patients undergoing non-SCS spine surgery, a setting in which leads, anchors, and the generator are especially likely to lie within or adjacent to the operative and electrocautery field. Across 58 surgical cases, documented management was infrequent at every phase. A perioperative plan appeared in the surgical or anesthesia preoperative note in 21 cases (36.2%), yet documented preoperative deactivation or surgery mode (Element 1) was present in only 8 (13.8%), documented reactivation before discharge in 11 (19.0%), and preoperative pain service or implanting physician consultation in 5 (8.6%). Only 2 cases (3.4%) met both prespecified elements.

Our findings align with, and offer a process-level explanation for, the downstream device failures reported by Norris and colleagues in 2023. In their single-surgeon series of 490 SCS implants, 15 generators (3%) were inactivated after a subsequent non-SCS surgery, 12 of which (80%) required surgical replacement, and surgery mode was frequently not activated beforehand [6]. We observed the upstream gap that can precede such events: deactivation or surgery mode was documented before incision in a minority of cases, and among monopolar electrocautery cases the return-pad location, one determinant of whether the current path traverses the generator, was not reliably documented. One patient in our cohort sustained generator damage requiring surgical replacement after monopolar cautery with an undocumented return-pad location. A single event cannot establish causation, but the sequence is consistent with the monopolar-electrocautery mechanism that Norris and colleagues proposed for their inactivations [6] and with the cautery precautions emphasized in current practical guidance [5].

Most of the events we observed are unlikely to reflect perioperative device management. Lead dislodgment, hardware exposure, and lead displacement occurred during revision or instrumented spine surgery with hardware in or adjacent to the operative field, and are recognized surgical risks that would not have been prevented by deactivation, surgery mode, or return-pad positioning. We report them because they describe the device-related burden in this population, not as evidence of management failure. Only one event, generator damage after monopolar electrocautery with an undocumented return-pad location, is mechanistically linked to the management elements we assessed.

This study has several limitations. First, our primary measures reflect what was documented. A device may have been turned off, placed in surgery mode, or managed appropriately without a corresponding note, so Element 1 in particular should be read as a lower bound on actual perioperative management. Second, this was a single-center retrospective study at one academic medical center, and documentation patterns and device-management workflows may differ elsewhere, limiting generalizability. Third, Element 2 captures only the laterality component of safe electrocautery practice, because return-pad-to-IPG distance was not reliably documented, and the laterality determination relied on interpretation of free-text operative descriptions of IPG and return-pad location; cases with no documented return-pad location were classified as nonadherent, a conservative choice that may underestimate alignment. Fourth, device-related adverse events were ascertained from the medical record, and events managed outside our system may have been missed. Patients who did not disclose their device, or whose stimulator was implanted in a spinal region remote from the operative site, may not have been captured or managed as device cases. In addition, later events (31 to 180 days) may be less plausibly attributable to the index surgery and are reported descriptively. Finally, the cohort size and low event rates preclude analysis of whether documentation or alignment was associated with device-related outcomes.

## 5 Conclusion

In this single-center retrospective cohort pilot study of patients with implanted spinal cord stimulators undergoing non-SCS spine surgery, documented perioperative device management was infrequent and fell well short of current practical guidance, with the largest gaps in preoperative deactivation or surgery-mode documentation and electrocautery management; only 2 cases (3.4%) met both prespecified elements. Whether these gaps reflect incomplete documentation, incomplete management, or both, they leave perioperative teams without the structured record that, for example, established cardiac-device pathways provide, and they coexisted with a small but non-trivial rate of device-related adverse events. Prospective evaluation of institutional protocols, structured documentation, and defined pain-service engagement is warranted to determine whether closing these gaps reduces device-related harm.

## Declaration of generative AI and AI-assisted technologies in the manuscript preparation process

During the preparation of this work, the authors used Claude (Anthropic) to assist with editing and formatting of the manuscript and with organizing and checking descriptive statistical output. The authors reviewed and edited the content as needed and take full responsibility for the content of the publication.
